# Characterisation of the dynamic nature of lipids throughout the lifespan of genetically identical female and male *Daphnia magna*

**DOI:** 10.1038/s41598-020-62476-z

**Published:** 2020-03-27

**Authors:** Julia K. Constantinou, Andrew D. Southam, Jouni Kvist, Martin R. Jones, Mark R. Viant, Leda Mirbahai

**Affiliations:** 1 School of Biosciences, University of Birmingham, Birmingham, B15 2TT UK; 2 Phenome Centre Birmingham, University of Birmingham, Birmingham, B15 2TT UK; 3 Research Program for Molecular Neurology, University of Helsinki, Haartmaninkatu 8, Helsinki, 00014 Finland; 4 Eawag, Swiss Federal Institute of Aquatic Science and Technology, Dübendorf, Switzerland; 5 Warwick Medical School, University of Warwick, Coventry, CV4 7AL UK

**Keywords:** Lipidomics, Ageing

## Abstract

Lipids play a significant role in regulation of health and disease. To enhance our understanding of the role of lipids in regulation of lifespan and healthspan additional studies are required. Here, UHPLC-MS/MS lipidomics was used to measure dynamic changes in lipid composition as a function of age and gender in genetically identical male and female *Daphnia magna* with different average lifespans. We demonstrate statistically significant age-related changes in triglycerides (TG), diglycerides (DG), phosphatidylcholine, phosphatidylethanolamine, ceramide and sphingomyelin lipid groups, for example, in males, 17.04% of TG lipid species decline with age whilst 37.86% increase in relative intensity with age. In females, 23.16% decrease and 25.31% increase in relative intensity with age. Most interestingly, the rate and direction of change can differ between genetically identical female and male *Daphnia magna*, which could be the cause and/or the consequence of the different average lifespans between the two genetically identical genders. This study provides a benchmark dataset to understand how lipids alter as a function of age in genetically identical female and male species with different average lifespan and ageing rate.

## Introduction

Lipids are essential for normal cellular functions including intracellular and extracellular signalling pathways, providing membrane fluidity and facilitating energy storage^1,2^. The importance of lipid molecules in cellular processes is reflected in the vast diversity of their structures and functions and thus they are involved in many homeostatic processes but also play a role in disease and long-term health^2^.

Model organisms have been crucial in enhancing our understanding of the role of different lipid classes in regulation of ageing rate, lifespan and prevention or acceleration of age-related diseases^3^. The first association between lipid composition and ageing rate was demonstrated in *Caenorhabditis elegans*^4^, *Drosophila melanogaster*^5^, laboratory *Mus musculus*^6^ and the budding yeast *Saccharomyces cerevisiae*. In *Saccharomyces cerevisiae*^7^, mutations in insulin growth factor-2 pathway and the mechanistic target of rapamycin complex 1 (mTORC1) signalling pathway increased lipid storage and the elevated lipid storage is associated with increased lifespan and reduced reproduction^3^. Therefore, there is a trade-off relationship between longevity, reproductive capacity and lipid storage. During reproduction, lipid reserves are used and depleted, while during low-reproductive periods lipid is accumulated, a phenomenon known as the *cost of reproduction*. Notably, animals with reduced reproduction often survive better, as energy from lipid reserves are invested into somatic maintenance and survival rather than reproduction^8^. In addition, in multiple species, including worms, flies, mice, rats, primates and spider mites, dietary restriction and change in concentration of lipid molecules (either as recorded blood levels or controlled dietary input), such as oleic acid can impact longevity ^3,9–14^. Using the yeast model organism *Saccharomyces cerevisiae*, it has been demonstrated that genetic and pharmacological interventions that can weaken but not completely inhibit metabolite flow through the pathway of *de novo* sphingolipid synthesis, can cause reduction in sphingolipid concentrations. The reduction in sphingolipid concentration can subsequently lead to increased replication and lifespan^3^. For example, deletion of Lag1 (homolog to human CERS2,) extends *S. cerevisiae* replication lifespan without altering the chronological lifespan. Whereas deletion of lsc1 (homolog to human nSMase 2) has been shown to shorten *S. cerevisiae* chronological lifespan^3^. This indicates a clear role for sphingolipids in regulation of replication and lifespan in yeast. Other lipids can regulate lifespan in *S. cerevisiae*, including triacylglycerides (TG). Excessive TG content is converted to ethanol which shortens chronological lifespan in yeast via an undetermined mechanism^3^. Therefore, caloric restriction (CR) can extend yeast lifespan by reducing the amount of excessive TG. Additionally, changes to the composition of mitochondrial membrane phospholipids has been linked to altered lifespan in yeast^3^. The changes in concentration of lipids and their impact on lifespan regulation and ageing process is a clear evidence of their significant role in regulation of health and disease. However, the relationship between lipids and longevity is complex and not all lipids within a class will have the same impact on lifespan^15^. The complex nature of this relationship can be demonstrated by the lipid class phosphatidylcholine (PC), where PC lipid species PC (O-34:1) and PC (O-34:3) have a negative association with diabetes and hypertension and a positive association with longevity^16^. Conversely, other PC species show either no effect or a negative effect with longevity^17^. This demonstrates the complex relationship between lipids, lifespan and healthspan.

In addition to lifespan, lipids can influence healthspan as the lipid composition and concentration directly affects cellular metabolism and function of a cell^17^. For example, lipids in membrane can influence many diverse biological functions, such as membrane fluidity and influence signalling capacity^18^. Membranes are also responsible for protecting enzymes and genes from external environments. However, with age the composition of lipids within a cell alters, including the membrane lipids^18^. This subsequently results in structural, functional and behavioural changes within a cell^18^, which can increase the development of age-associated diseases. As a result, certain lipid profiles can predict the risk of age-associated diseases, such as type 2 diabetes (T2D), cancer, and coronary heart disease^2,19^. In T2D - a common age-associated disease - a relationship has been established between increased risk of T2D and lower carbon number and double bond content of TG^20^. This relationship stands true for other lipids, including ceramides (Cer), phosphatidylcholines (PC) and sphingomyelins (SM)^20^. Such findings begin to answer the question as to why dyslipidemia (the presence of an abnormal amount of lipids) is an independent risk factor of T2D^20^. Furthermore, TG and cholesterol are used to determine risk of cardiovascular diseases^17^ while TGs and lipoprotein levels have been linked with increased risk of obesity and hence can negatively impact health and longevity^1^. Altered lipid metabolism is also a prominent feature of cancer due to increased demand to support accelerated proliferation^21^ with altered levels of TG specifically associated with many types of cancer^22–24^. These data provide clear support for role lipids in regulating healthspan in many species.

Most interestingly, it is well-established that females and males of various species have different average lifespan, healthspan and ageing rate^25^. However, the molecular pathways that contribute to these observed differences between male and females are not fully understood. As lipids clearly play a vital role in regulation of ageing rate, healthspan and lifespan^26^, it is plausible that they may also contribute to the differences observed in ageing rate between the two sexes. Indeed, the lipid composition of male and females are different^27–30^. This is partly, due to different reproduction, hormonal and growth needs of both sexes. Furthermore, several studies have demonstrated that there are clear sex associated differences in lipid profile with age^31–35^. However, further detailed studies are required to enhance our understanding of the role of lipids in regulation of lifespan between sexes. Short-lived model organisms with different average lifespan between females and males can help to elucidate the molecular mechanisms of lifespan and healthspan regulation between sexes.

*Daphnia magna* are a recognised model organism by the U.S. National Institute of Health^36^. They have been extensively used across a variety of research fields including ecotoxicology, ecology and population genetics and is ever-growing in importance for molecular studies of neurobiology and the biology of ageing^37^. Importantly, *D. magna* reproduce via cyclical parthenogenesis whereby mothers produce genetically identical daughters. During unfavourable conditions or through hormonal stimulation, male *Daphnia* are produced for sexual reproduction. Both females and males are genetically identical yet demonstrate different average lifespans, body size, swimming speed and ageing rate^37^. These observations bring *D. magna* to the forefront of alternative models for research into ageing. Therefore, in this study we used genetically identical female and male *D. magna* to gain a comprehensive understanding of how their lipid composition alters as function of age and sex. To capture the dynamics nature of lipids as a function of age and sex, we conducted a detailed time course lipidomics study with 90 samples covering 15 female age groups and 12 male age groups. The information can reveal how alteration to lipid profiles or lipid signalling influence longevity overall and provide greater insight into sex differences in longevity. It will also improve our understanding of inherited pro-longevity lipid profiles and help elucidate mechanisms behind lipid metabolism and it’s interaction with lifespan extension^1^. Overall, the magnitude of this study, alongside using genetically identical female and male species that have evolved different average lifespan and ageing rate^37^, makes this study unique and extremely powerful in regards to advancing our understanding of the molecular regulation of lifespan between sexes.

## Experimental Procedures

### *Daphnia magna* stocks and maintenance

Cultures of *Daphnia magna* Bham 2 strain^38^ have been maintained in the laboratory conditions at the *Daphnia* Facility, University of Birmingham, UK for over 10 years as previously described^37,39,40^. In this work, *D. magna* Bham 2 strain were maintained in photoperiodic lighting (16 h of light: 8 h of dark) and temperature of 20 ± 1 °C, in high hardness COMBO medium minus EDTA^41^,. Animals were fed every other day with *Chlorella vulgaris* at a concentration of ≈27,550 cells of algae per individual *Daphnia*. Media was changed three times a week and cultures maintained at a maximum of 1 adult/50 ml of media at a minimum density of 5 individuals/250 ml.

### Chemical-induction of genetically identical male *D. magna* BHAM 2

Female *D. magna* Bham 2 strains were treated with the crustacean reproductive hormone, (E,E) Methyl Farnesoate (MF; Tebu-Bio ltd, Peterborough, UK) according to the protocol described in our previous publication^37^. Briefly, male *Daphnia* were induced by exposing sexually matured individual female *Daphnia* (age of maturity for *D. magna* Bham 2 strain: 9 days old) to MF at a final concentration of 400 nM. Due to the instability of MF, media was changed daily to ensure consistent exposure. The first brood was discarded and male neonates were collected from 2^nd^–5^th^ broods. Female and male cultures were maintained separately.

### Sample collection

**Figure 1:**
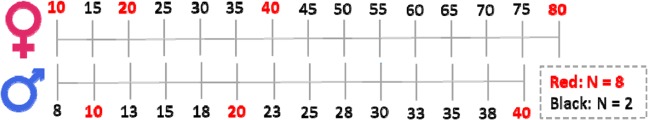
Sample collection details for female and male *D. magna*. Sample ages for females are shown at the top where the minimum sampled age is 10 days and the maximum sampled age is 80 days. Male sample ages are shown on the bottom and have a minimum sampled age of 8 days and maximum sample age of 40 days. Ages shown in red have a biological replicate number of 8, ages shown in black have a biological replicate number of 2.

Samples were collected at regular intervals for female and male *D. magna*, as shown in Fig. 1.

When male *D. magna* had reached the sampling age, samples were collected at the same time of day for all sampling groups, washed with deionised water and flash frozen in liquid nitrogen. Female samples were collected using the same method, however, to ensure females were in a similar reproductive stage and no signal would be detected from neonates, individuals with empty brood pouches were collected within a 2-hour window of brood release.

### Sample preparation for lipidomics analysis

To ensure the same amount of biomass was used for lipid extractions regardless of the age and size of the *Daphnia*, an initial experiment was conducted to measure the dry mass of male and female *Daphnia* at different ages and calibration curves were created. This enabled the used of consistent biomass (equivalent to dry mass 4.37 mg) for each lipid extraction for all sample groups (Supplementary Figure S1a,b; Đức, *et al*.^42^). Lipid extractions were conducted using a biphasic approach (chloroform/methanol/water; ratio – 2/2/1.8) as described in detail by Wu, *et al*.^43^ and Mirbahai, *et al*.^44^. Extract blanks were produced following the same protocol without *Daphnia* material. Lipid extracts equivalent to 4.73 mg biomass were dried under a stream of nitrogen gas.

### Sample resuspension for ultra-high performance liquid chromatography mass spectrometry (UHPLC-MS) lipidomics

Ice-cold 1:1 acetonitrile: propan-2-ol (80 µl; Fisher Chemicals: LC/MS grade A955-212, LC-MS grade A461-212) was added to the dried extracts and vortexed (30 s). Equal amount (15 µl) was taken from each sample, pooled and vortexed to create the intrastudy quality control (QC) sample. Samples were centrifuged (4 °C, 4000 rpm, 10 minutes). Supernatant (55 µl) was transferred to a low-volume HPLC vial (Thermo Fisher) and stored on ice. The QC sample was vortexed and centrifuged (4 °C, 4000 rpm, 10 minutes) and divided into 23 aliquots of 50 µl and loaded into low-volume HPLC vials. Vials were placed in an autosampler (7 °C).

### UHPLC-MS/MS lipidomics

UHPLC-MS lipidomics is a semi-quantitative approach that identifies relative fold-changes of lipids across different phenotypic groups^45^. Lipidomics data were acquired using a Dionex UltiMate 3000 Rapid Separation LC system (Thermo Fisher Scientific, MA, USA) coupled with a heated electrospray Q Exactive mass spectrometer (Thermo Fisher Scientific, MA, USA). Extracts were analysed on a C30 column (Accucore 150 × 2.1 mm, 1.7 µm; Thermo Fisher Scientific, MA, USA). Mobile phase A consisted of 10 mM ammonium formate in 50% v/v acetonitrile/water (Fisher Chemicals, A955-212) with 0.1% formic acid and mobile phase B consisted of 2 mM ammonium formate in 10:88:2 v/v/v acetonitrile, isoproponyl (Fisher Chemicals, A461-212) and water (Merck, 1.15333.2500) with 0.02% formic acid. Gradient: t = 0.0, 22% B; t = 6.0, 60% B; t = 14.0, 85% B; t = 23.0, 100% B; t = 26.0, 100% B; t = 26.1, 22% B; t = 30.0, 22% B. All changes were linear (curve = 5) and the flow rate was 0.40 mL/min. Column temperature was 45 °C and injection volume was 5 μL. Data were acquired in both positive and negative ionisation UHPLC-MS/MS within the mass range of 200–1800 m/z at resolution 70,000 (full scan data; FWHM at m/z 200) and 35,000 (MS/MS data). The UHPLC-MS/MS scan cycle was as follows: full scan, 5xHCD MS/MS (top 5 most intense ions), repeat. An MS/MS exclusion list - created from an extract blank sample was used in the method. Stepped normalised collision energies of 25 and 30 were used, and dynamic exclusion was set to 8 s. Ion source parameters were set as follows: sheath gas = 60 arbitrary units, aux gas = 20 arbitrary units, sweep gas = 1 arbitrary units, spray voltage = 3 kV capillary temp. = 285 °C, aux gas heater temp. = 370 °C. Automatic gain targets of 1e6 (full scan MS) and 1e5 (MS/MS) were used. The purpose of the MS/MS protocol detailed here was to fragment as many of the detected peaks as possible to allow them to be annotated by comparison to MS/MS libraries, described below.

### Data analysis and lipid annotation

LipidSearch (version 4.2, Thermo Fisher Scientific) was used to de-convolute and integrate peaks in the data, and annotate peaks based on their MS/MS fragmentation patterns. In terms of deconvolution, this software generated two data intensity matrices – one for positive and one for negative ionisation modes – containing samples as rows and annotated lipids as columns. Only lipids that were annotated from their MS/MS spectrum were retained in the datasets. For lipid annotation, all experimental LC-MS/MS spectra data were searched against a MS/MS lipid library in the LipidSearch software database. The generated MS/MS fragmentation spectrum can be used to identify the class and fatty chain composition of lipid species (some examples of MS/MS spectra from lipid species measured in this study are shown in Supplementary Data S1: Section B.). Experimental MS/MS spectra were searched against all lipid classes in the LipidSearch database using the following potential ion forms: positive ion = [M + H]^+^, [M + NH_4_]^+^, [M + Na]^+^, [M + K]^+^, [M + 2 H]^2+^; negative ion = [M-H]^−^, [M + HCOO]^−^, [M + CH_3_COO]^−^, [M + Cl]^−^, [M-2H]^2−^. The quality of the annotation was graded as A-C. This is defined as: Grade A = all fatty acyl chains and class were completely identified; Grade B = some fatty acyl chains and the class were identified; Grade C = either the lipid class or some fatty acyls were identified. Only peaks that had an annotation grade of A-C were retained in the dataset. Peaks in the data matrices were then filtered as follows: (i) peaks that were present in both the QCs and the extract blank with a extract blank:QC intensity ratio of >10% were removed; (ii) samples with >20% missing values were removed; (iii) peaks with a median QC intensity RSD > 30% were removed; (iv) matrix was normalised by PQN^46^ and (v) missing values were filled by KNN^47^. R (R Project for Statistical Computing, http://www.r-project.org/, Version 3.5.2) was used for all statistical analyses and data visualisation. First, positive and negative data matrices were checked to ensure no overlapping ion forms were detected. The datasets were concatenated and treated as a single dataset for the remainder of the analysis. Principal component analysis (PCA) was applied and visualised using package ggplot2^48^. Data was analysed by the R package ‘Limma’^49^ with the core capability of log2 transforming data then using linear models to assess differential expression in the context of multifactorial experiments^50^. Empirical Bayesian methods were then applied to smooth standard error to provide stable results for lipids in conditions age only (irrespective of sex), sex only (irrespective of age) and sex:age interaction. A further two results files were subsequently generated for the condition of age in females only and another for males only.

Venn diagrams, barplots and heat map were produced using R package ‘ggplot2’ and the results tables produced via Limma. For the barplots, significantly changing lipids showing an increasing trend or decreasing trend were selected by adjusted *p-*value <0.001 with a log fold change of <0 and >0, respectively. The method of adjustment applied was BH as described by Benjamini and Hochberg^51^. For heatmaps analysis, the results datasets (sex, ageF, ageM, sex:age interaction) were ordered by adjusted *p-*value and the top 50 most significantly changing lipids from each ion mode were selected for heatmap visualisation.

## Results

### Age and sex influences lipid profile in *D. magna*

**Figure 2:**
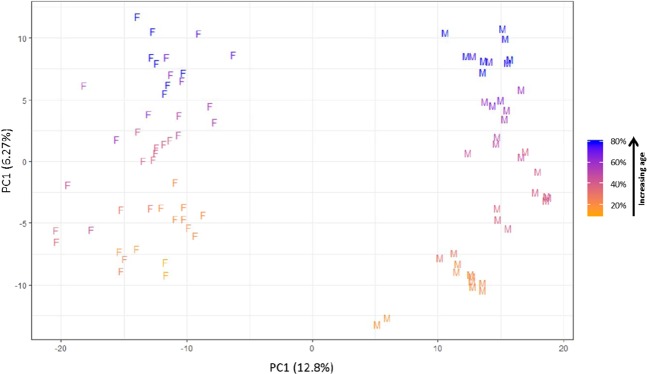
Principal component analysis of normalised positive and negative ionisation mode lipidomics data for male and female *Daphnia magna* demonstrated as percentage of lifespan. Principal component 1 shows clear separation based on sex (F = female, M = male) with females grouped to the left and males to the right. Principal component 2 shows separation by age depicted by colour ranging from orange (young) to blue (old). Female *D. magna* age range is 10 days to 80 days (maximum sampled age = 80 days). For female age groups 20, 40 and 80 days, eight technical replicates were used and for intermediate ages two technical replicates were used. Male *D. magna* age groups ranging from 8 days to 40 days were used (maximum sampled age = 40 days). For male *D. magna* age groups 10, 20 and 40 days, eight technical replicates were used and for intermediate age groups two technical replicates were used. For detailed information regarding biological replicate numbers see Supplementary Tables S1 and S2. Supplementary Figures S5 and S6 show PCA plots for positive and negative ion mode separately and highlight the grouping of the intrastudy QC sample.

PCA was conducted on the normalised combined negative and positive ion mode lipidomics dataset. As demonstrated in Fig. 2, *D. magna* samples were separated along the principal component 1 (PC1) according to sex (with no overlap between male and female groups). Samples were further separated along the PC2 axis according to age, from young (orange) to old (blue).

Based on the PCA results, it was prudent to analyse the data in terms of sex, female ageing, male ageing and sex:age interaction. Throughout the analysis, it became apparent that not all lipid sub-species within a single class behave in the same way. Table 1 highlights 10 lipid sub-species and their associated class, ordered by most to least significantly changing relative intensity in the condition of sex:age interaction, with the response of the same lipid sub-species for the conditions of sex, female ageing and male ageing. Table 2 shows some reported biological functions related to the classes of the 10 lipids included in Table 1. The details for all annotated lipids are reported in Supplementary Table S3.

**Table 1:** Top 10 lipids with most significantly changing relative intensity in sex:age interaction plus response of the same lipids in sex, ageing in females only and ageing in males only.

DESCRIPTION		SEX:AGE INTERACTION			SEX ONLY			FEMALE AGE ONLY			MALE AGE ONLY		
LipidIon	Class	logFC	AveExpr	adj.P.Val	logFC	AveExpr	adj.P.Val	logFC	AveExpr	adj.P.Val	logFC	AveExpr	adj.P.Val
SM(t40:2)+HCOO	SM	−0.1068	−1.6318	3.24E-21	1.9720	−1.6318	1.96E-13	0.0025	−1.3352	0.36712	−0.1042	−1.9647	7.53E-15
ChE(16:1)+NH4	ChE	0.1241	3.8752	1.55E-20	−3.4721	3.8752	1.67E-18	−0.0423	3.7010	2.19E-11	0.0825	4.0706	5.74E-15
SM(t40:2)+H	SM	−0.0675	2.4276	1.83E-20	1.2463	2.4276	1.08E-11	−0.0032	2.5644	0.09941	−0.0707	2.2741	1.29E-15
TG(2:0_14:0_16:3)+NH4	TG	0.0583	3.6851	1.89E-20	−1.0510	3.6851	4.47E-10	0.0166	3.6932	2.72E-09	0.0752	3.6759	8.65E-25
SM(d34:3)+H	SM	−0.0710	3.7734	3.59E-20	1.5936	3.7734	1.07E-14	−0.0027	3.7889	0.23171	−0.0733	3.7560	5.39E-16
TG(2:0_16:0_16:3)+NH4	TG	0.0459	6.3280	8.82E-20	−0.6185	6.3280	2.08E-06	0.0171	6.2759	1.51E-11	0.0633	6.3864	9.17E-26
ChE(18:1)+NH4	ChE	0.0945	6.3903	1.50E-19	−4.5301	6.3903	1.71E-31	−0.0356	7.1095	7.51E-14	0.0597	5.5834	1.38E-12
ChE(17:1)+NH4	ChE	0.1042	1.0156	1.66E-19	−3.8441	1.0156	5.71E-24	−0.0410	1.2567	1.23E-14	0.0632	0.7451	1.34E-10
TG(16:0_16:0_18:3)+NH4	TG	0.0165	12.7314	2.84E-19	−0.1669	12.7314	0.00035	0.0040	12.6646	9.19E-12	0.0205	12.8064	4.94E-16
TG(36:4)+NH4	TG	0.0491	7.880	6.88E-19	−0.6493	7.8801	4.74E-06	0.0158	7.7948	2.64E-09	0.0647	7.9759	5.30E-24

Lipids were ordered by adjusted p-value from most significant to least for the condition of sex:age interaction. For the conditions of sex only, ageing in females only (female age only) and ageing in males only (male age only), lipids were ordered to match that of sex:age interaction. Log fold-change (logFC), average expression (AveExpr) and adjusted p-value (adj.P.Val) were recorded for each condition. Where average expression is positive: for sex:age interaction this is changing at a faster rate in females; for sex only this is higher in females; in female ageing only this is increasing with age; in male ageing only this is increasing with age. The MS/MS fragmentation spectra of all lipids listed in Table 1 have been manually reviewed (Supplementary Data S1: Section B).

**Table 2:** Biological function of lipid classes included in Table 1.

Class	Biological Function	Reference
SM	SM facilitates formation of lateral membrane domains and has a strong interaction with cholesterol. SM has a varied and vast biological influence including but not limited to; regulation of endocytosis and receptor-mediated ligand uptake, ion channel and G-protein coupled receptor function, protein sorting, and functioning as receptor molecules for various bacterial toxins, and for non-bacterial pore-forming toxins.	^76^
ChE	ChE is the inactive form of cholesterol and it is primarily used for transport of cholesterol to organs or to act as a biologically inert storage of excess cholesterol by the addition of fatty acids to the hydroxyl group results in less polarity making the lipid ‘inert’	^71^
TG	TGs are the main form of fat recorded in humans and are present across many species. They are found in the blood and act as an energy supply for the body in addition to being stored as body fat when excess is present.	^77,78^

The top 10 lipids with most significantly changing relative intensity in sex:age interaction were lipid sub-species of three lipid classes; SM (sphingomyelin, ChE (cholesterol ester) and TG (triglyceride).

### Overview of changes in lipidomics of male and female *D. magna* as a function of age and sex

**Figure 3:**
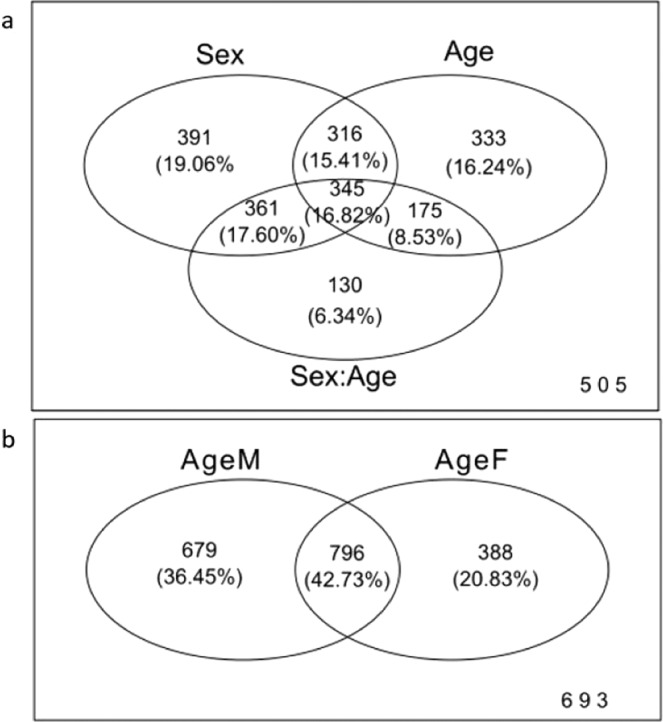
The Venn diagram represents an overview of the number of lipids with significant change in their relative intensity between categories of sex, age and sex:age interaction, and ageing in males and females in *D. magna*. (**a**) The number of annotated lipids found to have a statistically-significant change in relative intensity (adjusted *p-*value <0.001) across the factors sex, age, and sex:age interaction are represented by numbers and the respective percentage within each grouping. The 505 lipids outside of the groupings do not show significantly (adjusted p-value <0.001) changing relative intensities in any of the conditions presented. (**b**) The number of lipids identified as showing a significant change in relative intensity (adjusted *p-*value <0.001) observed within the conditions of ageF (female ageing data only) and ageM (male ageing data only) are represented by numbers and percentages within each grouping.

Of the total 2,556 annotated lipids (based on their MS/MS fragmentation pattern) used for the subsequent analysis, 2,051 lipids showed significantly altered relative intensity (FDR adjusted *p*-value <0.001) as a function of sex (1,413), age (1,169) or sex:age interaction (1,011) (Fig. 3a, Supplementary Table S3). The significantly altered lipids are referred to as ‘significant annotated lipids’ for the remainder of the paper.

Roughly an equal number of significant annotated lipids are changing as a function of age (1,169) and are different between the sexes (1,413). Interestingly approximately half (1,011) of all significant annotated lipids are interacting between age and sex. A fraction of lipids (345) are changing as a function both sex and age (reported to change significantly as a function of sex and age), and also have significant interactions between age and sex (have different age-related changes in the two sexes). Lipids that are significant for only one variable are rare, especially for sex:age interactions. Only 27.67% (391/1,413) of sex-dependent changes show no age-related changes or sex:age interactions, meaning these lipids do not change during ageing, but are different between male and female *Daphnia*. A similar number of lipids (28.49%; 333/1,169) with age-related changes were equal in both sexes. While only 12.86% of sex:age interaction significant lipids had no significant changes for age or sex, meaning there are fewer lipids where the independent effects of sex and age is cancelled out. For example, when the lipid amount increases in one gender during ageing and at the same time decreases with age in the other gender with equal magnitude, the overall effect is cancelled out in an interaction setting. The identity of the lipids that alter as a function of age or sex can provide valuable information regarding the role of lipids that drive the differences observed in ageing rate between the two sexes.

As shown in Figs. 3a, 16.82% of the significant annotated lipids are shared between all three categories (sex, age or sex:age interaction), meaning these lipids have age-related changes in both sexes, but also the rate of change is different between the sexes. Interestingly, 15.41% of the significant annotated lipids are shared between age and sex groups and not present in sex:age interaction group. These lipids are changing the same way in both sexes during ageing, but there is also a basal difference in the lipid levels between the sexes.

### Age-dependent changes in lipid profile of female and male *D. magna*

When analysing age-associated changes in relative intensity of lipids in the context of ageing in female only (AgeF) and ageing in male only (AgeM), a total of 2,556 lipids were identified, of which 1,863 were expressing significantly altered lipid relative intensity (<0.001 adjusted *p*-value), see Supplementary Table S3. The lipids found to have statistically significant changes in relative intensity with ageing are referred to as ‘age-related annotated lipids’.

As shown in Fig. 3b, a high percentage of annotated lipids (42.73%) alter as function of age and independent of sex in *D. magna*. Interestingly, there are more unique age-related annotated lipids changing in male only samples (36.45%) compared to female only samples (20.83%).

**Figure 4:**
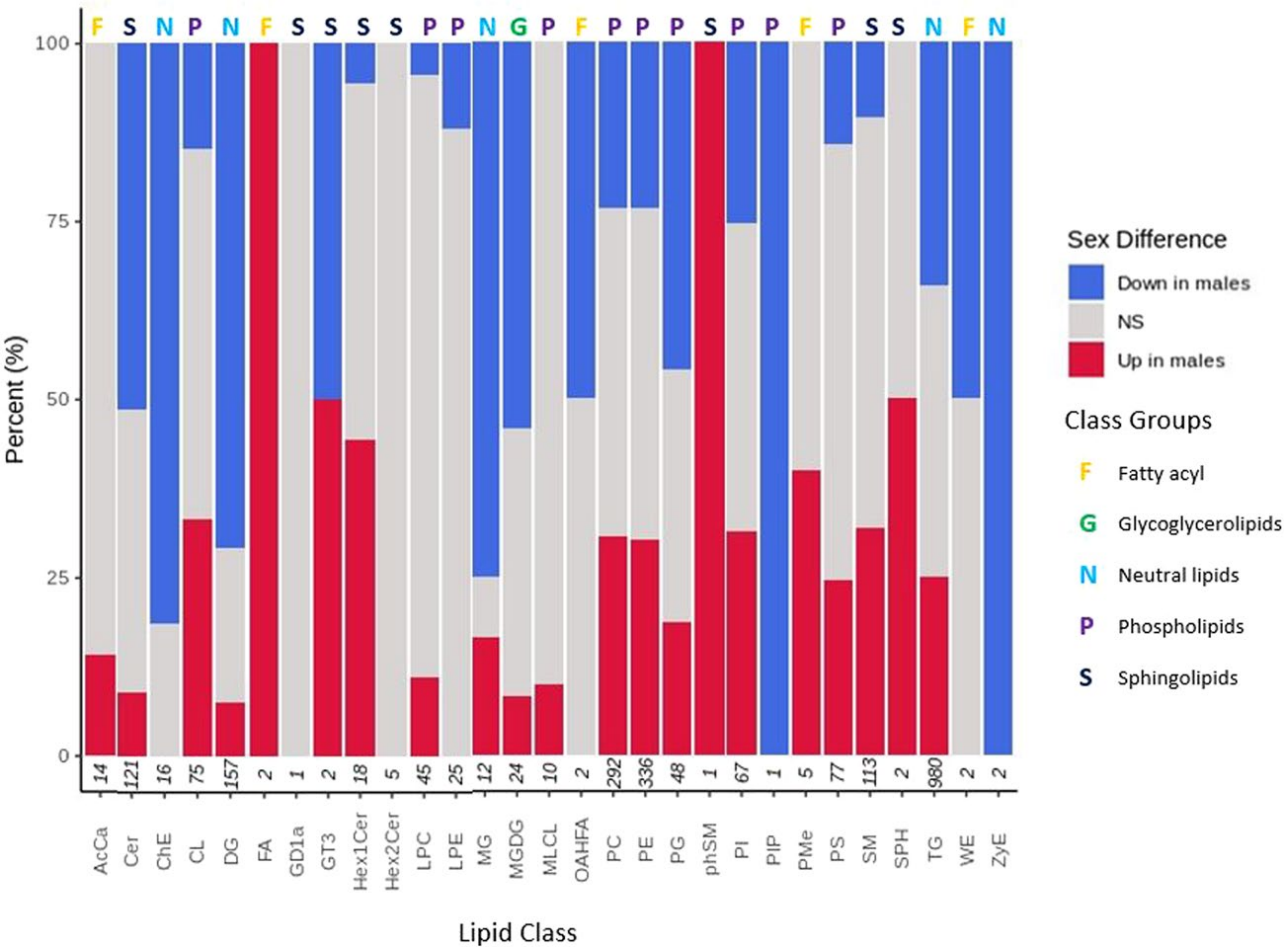
Differences in lipid class between male and female *D. magna*. Stacked normalised bar chart shows percentage of lipid classes with statistically-significant changes between sexes. Lipids lower in males are shown in blue whilst lipids higher in males are shown in red. Grey represents no significant difference between sexes (adjusted *p*-value <0.001). For female age groups 20, 40 and 80 days eight technical replicates were used and for intermediate age groups two technical replicates were used. For male *D. magna* age groups 10, 20 and 40 days eight technical replicates were used and for intermediate age groups two technical replicates were used. For detailed information regarding biological replicate numbers see Supplementary Tables S1 and S2. Numbers on the x-axis represent the total number of lipids identified in each class. Numbers on the x-axis represent the total number of lipids identified in each class, for lipid class abbreviation descriptions and classification see Supplementary Table S7.

LipidSearch 4.2 was used, as described in the method section, to identify the categories of significantly altering lipids as a function of sex. The identified lipids are categorised into five over-arching class groups as shown in Fig. 4 and listed in Supplementary Table S7.

Within these six over-arching groups, certain sub-classes of lipids are more common in the overall dataset. For example, triglycerides (TG) and diglycerides (DG) account for 82.26% and 13.45% of the total neutral lipids and sterols. Phosphatidylethanolamine (PE) and phosphatidylcholines (PC) account for 34.47% and 29.92% of glycerophospholipids, respectively. Within sphingolipids, ceramides (Cer) and sphingomyelin (SM) account for 46.01% 42.97% of the lipids, respectively. Some MS/MS fragmentation spectra for species within each of the 6 over-arching classes (plus some other examples) were manually checked to confirm that the identifications generated by the automated annotation software were correct (Supplementary Data S1: Section B).

Most interestingly, the sub-classes behave differently between sexes as seen in Fig. 4 and Supplementary Table S4. For example, the level of TG (34% lower) and DG (70.70% lower) neutral lipids are generally lower in males compared to females. Likewise, the level of lipids categorised as sphingolipids are largely lower in males, as shown by 51.24% of ceramides demonstrating a lower amount in males and only 9.09% are higher in males compared to females. In contrast, the level of certain lipid sub-classes are generally higher in males than females, including SM, with 31.86% of SM demonstrating a higher amount in males than in females. Finally, there are lipid categories that display no significant difference between sexes, such as GD1a and Hex2Cer. The total number of lipids classified as within these groups occur at much lower frequencies compared to other classes (Fig. 4).

### Accumulative change in the levels of lipids in female and male *D. magna* with age

**Figure 5:**
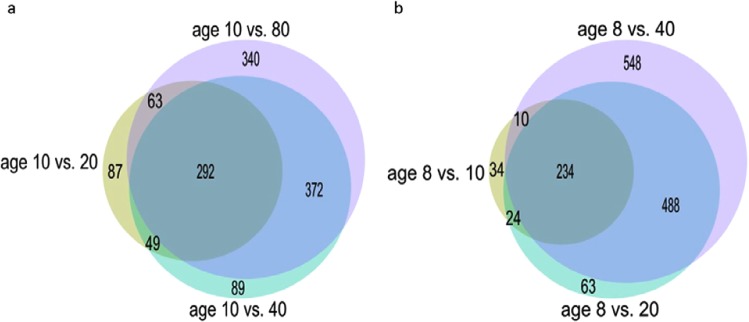
Venn diagram of lipids statistically-significantly changing lipid intensity with age between increment age groups compared to youngest age group for each gender. (**a**) Comparison of lipids in female *D. magna* showing statistically-significant changes in lipid intensity (*p-*adjusted <0.001) from age groups 20, 40 and 80 days compared to youngest female group aged 10 days shows larger difference with increased age (e.g. between 10 and 20 days only 87 unique lipids identified showing similarity between age groups compared to 340 unique lipids between aged 10 days and 80 days). The size of the circle is proportionally relative to number of lipids. (**b**) Comparison of significant lipids in male *D. magna* (*p-*adjusted <0.001) from age groups 10, 20 and 40 days compared to youngest male group aged 8 days shows larger difference with increased age (e.g. between 8 and 10 days only 34 unique lipids identified showing similarity between age groups compared to 548 unique lipids between aged 8 days and 40 days).

Figure 5a highlights the statistically-significant changes in lipid intensities across the main age groups of female *D. magna*; ages 20, 40 and 80 days compared to the youngest age group of 10 days old. When using age 10 days as the reference group for statistical comparisons, a clear statistically significant trend of change in intensities of lipids is observed between young (10 days old) and older (20, 40 and 80 days old) female *Daphnia*. Only 87 lipids are unique to aged 10 days compared to aged 20 days (age 10 vs 20), indicating relatively few differences between the lipidomes of these two age groups. By contrast, 340 lipids were found to have statistically-significant intensity differences when age groups 10 and 80 days were compared (age 10 vs 80) highlighting greater disparity between the lipidomes of these two age groups than between the 10 and 20 day groups. This clearly demonstrated that there is a gradual divergence of the lipidome over time leading to larger difference between the oldest age group of 80 days compared to aged 10 days. A similar trend can be observed when using females aged 20 days as the reference group (Supplementary Table S5). Interestingly, at age 40 days old (approximately 40% of lifespan for female *D. magna*) there is 118 (4.62%) and 242 (9.47%) lipids with significantly altered lipid intensities compared to age 10 days and 80 days, respectively (Supplementary Table S5). This data also provide evidence that the changes in lipid profile of *D. magna* as a function of age are accumulative and occur gradually throughout the lifespan of *Daphnia* rather than rapidly occurring at a specific time in the lifespan of *Daphnia*.

Finally, when using females aged 80 days as the reference group (Supplementary Table S6), a similar trend to when using age 10 days old *Daphnia* was observed, supporting a continuous divergence of the lipidome with age.

The same trend observed in females is also observed in male *D. magna* whereby there is less difference between young age groups (e.g 8 and 10 days) and a larger difference between young and old age groups (e.g 8 and 40 days; see Fig. 5b, Supplementary Table 6). The main difference between sexes is that when 40% of lifespan in males (aged 20 days) is used as the reference, the range of results are much wider with 89 (3.48%) lipids comprising the smallest group (comparing 20 days v 8 days and 20 days v 40 days) and 428 (16.74%) lipids representing the largest group (20 days v 40 days). The 3.48% lipids uniquely altered in intensity in aged 20 days v 8 days, compared to aged 20 days v 40 days shows that the majority of lipids changing between 8 and 20 days are the same lipids continuing to significantly change between aged 20 days and 40 days, with only 3.48% difference. Overall the lipidome in ageing males appears to follow the same trend as seen in females with a gradual divergence over time.

**Figure 6:**
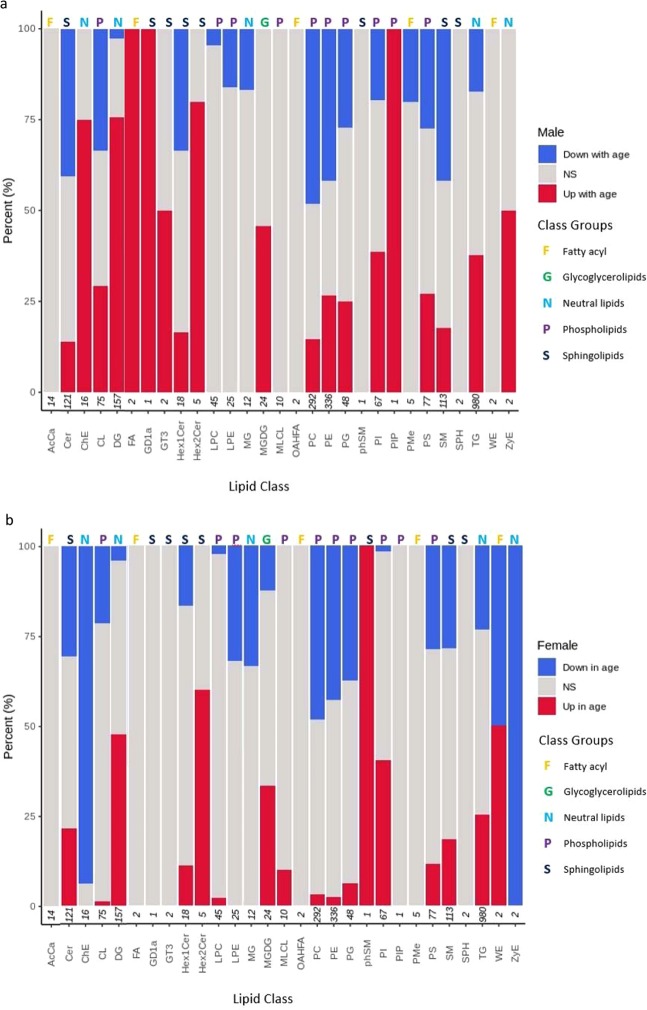
Directional change of lipid levels significantly changing in (**a**) female and (**b**) male *D. magna*. Stacked normalised bar chart shows percentage of lipid classes that are changing with age in *D. magna*. Lipids showing overall decline with age are shown in blue whilst lipids showing overall increase with age are shown in red. Grey represents no significant change with age (adjusted *p*-value <0.001). Numbers on the x-axis represent the total number of lipids identified in each class, for lipid class abbreviation descriptions and classification see Supplementary Table S7. For female age groups 20, 40 and 80 days eight technical replicates were used and for intermediate age groups two technical replicates were used. For detailed information regarding biological replicate numbers see Supplementary Table S1. For male age groups 10, 20 and 40 days eight technical replicates were used and for intermediate age groups two technical replicates were used. For detailed information regarding biological replicate numbers see Supplementary Table S2.

Certain lipid sub-classes behave differently (i.e. change in their relative intensity level) with age between female and male *D. magna* samples, leading to a different lipid profile between male and females as function of age (Fig. 6a,b). For example, while the relative intensity (i.e. level) of equal number of lipids in the PE, PC and DG sub-categories decreases with age in both females and males, higher percentage of lipids in these sub-categories show an increase relative intensity as a function of age in males (PE: 26.79%, PC: 14.73%, DG: 75.80%) than females (PE: 2.38%, PC: 3.08%; DG: 47.77%). Other clear differences between females and males is detected in the Cer and SM sub-categories. Higher percentage of lipids in these sub-categories show a decrease in their relative intensity as a function of age in males (Cer: 40.50%, SM: 41.59%) than females (Cer: 30.58%, SM: 28.32%). While the opposite trend is observed for the TG sub-category with higher percentage of lipids in this sub-category demonstrating a decrease in their relative intensity as a function of age in females (TG: 23.16%) than in males (TG: 17.4%). Most interestingly, there are sub-categories that only alter in their relative intensity as a function of age in one of the sexes while remaining unchanged in the other sex. For examples, within the 16 ChE lipids, 93.75% decline with age in females yet 0% decline with age in males. For the same lipids, 75.00% increase with age in males and 0% increase in females.

### Sex:age interaction

**Figure 7:**
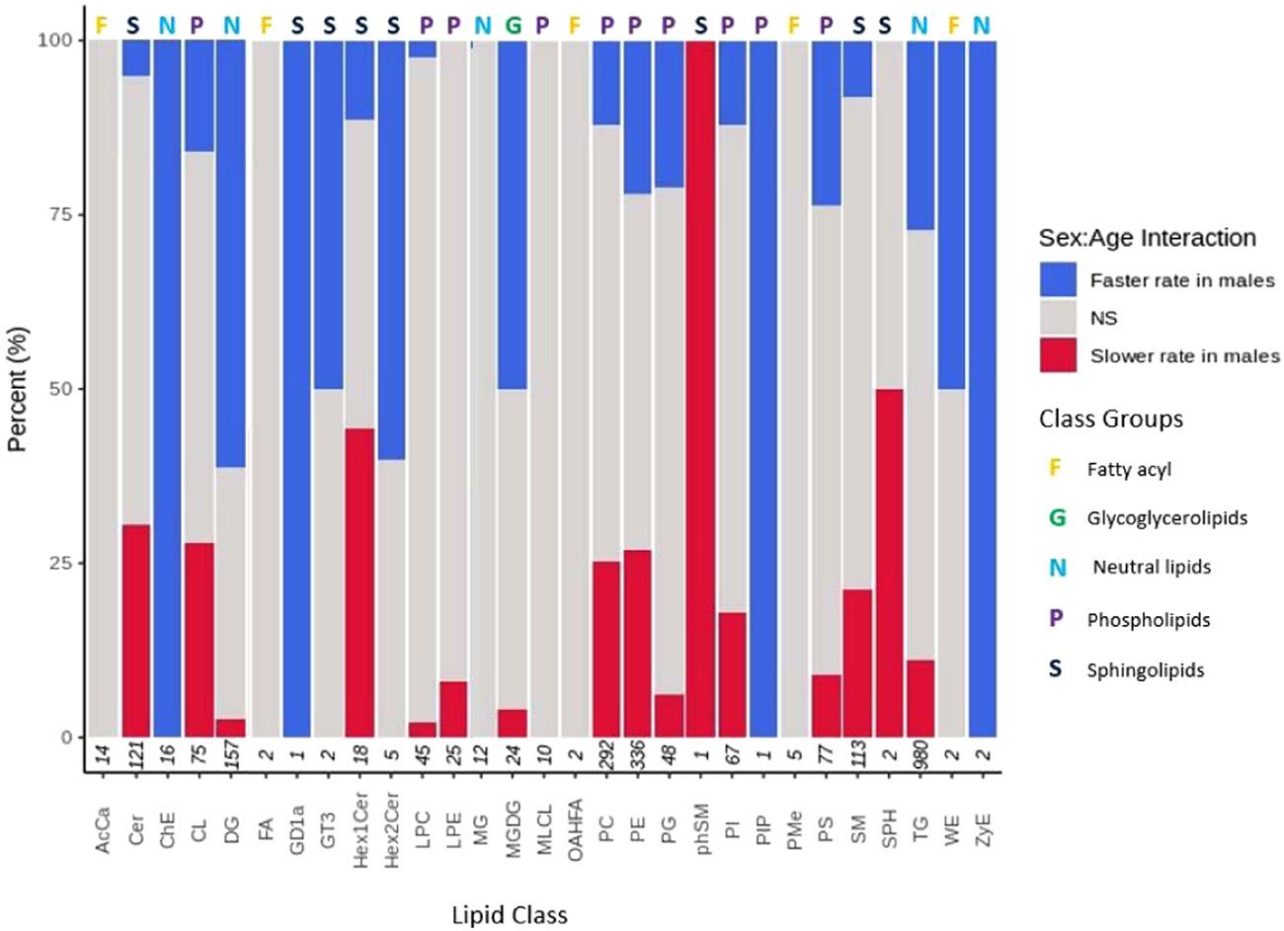
Lipid class changes in sex:age interaction in male and female *D. magna*. Stacked normalised bar chart shows percentage of lipid classes that are changing with sex:age interaction. A faster rate of change in males with age is shown in blue whilst slower rate in males with age (faster rate in females) is shown in red. Grey represents no significant change with interaction between sex and age (adjusted *p*-value <0.001)). For female age groups 20, 40 and 80 days eight technical replicates were used and for intermediate age groups two technical replicates were. For male *D. magna* age groups 10, 20 and 40 days eight technical replicates were used and for intermediate age groups two technical replicates were used. For detailed information regarding biological replicate numbers see Supplementary Tables S1 and S2. Numbers on the x-axis represent the total number of lipids identified in each class. Numbers on the x-axis represent the total number of lipids identified in each class, for lipid class abbreviation descriptions and classification see Supplementary Table S7.

Sex:age interaction group represents differential rate of change during ageing in the two sexes in the relative intensity of the lipids. The concentrations of different lipids in different sub-classes alter at different rates as a function of age between females and males (Fig. 7). For example, the concentrations of 26.94% and 61.15% of the lipids in the sub-classes of TG and DG, respectively, alter at a higher rate in males compared to females. On the other hand, the concentration of 30.58% and 21.14% of lipids in the ceramides and sphingomyelin sub-classes, respectively, alter at a much slower rate in males compared to females with age.

The differential rate of change of some lipid intensities with age appear to show significance in one gender, for example either reportedly changing at a faster rate in males or no significant change (no lipid sub-species are seemingly changing at a faster rate in females). Of the 34 lipids that change in a single direction only, 29.41% express a faster rate of change in males. Contrastingly, only 5.88% are changing at a slower rate in males (faster rate in females) and 14.70% have no significant change in either direction.

**Figure 8:**
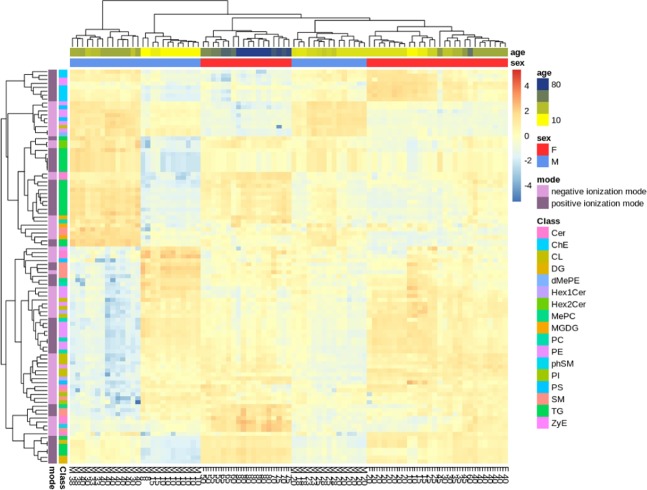
Heatmap of lipid changes with sex:age interaction. Heatmap of lipidomics analysis for top 50 lipids significantly-changing concentrations in positive ion mode (dark purple) and top 50 lipids significantly changing levels in negative ion mode (light purple, adjusted *p*-value <0.001) in *D. magna*. The heat map shows 100 selected lipids indicated by class colours (rows) and 29 samples (columns). Colours correspond to per-lipid z-score that is computed from area under the curve. Lipids and samples were hierarchically clustered based on Euclidean cluster extraction and average linkage. Numbers on the x-axis represent the total number of lipids identified in each class, for lipid class abbreviation descriptions and classification see Supplementary Table S7.

Figure 8 is the heatmap representation of the intensity levels of the top 50 lipids, in both negative and positive ion modes, with the most significant adjusted *p*-value in their intensity as a function of interaction between age and sex. As shown in this figure, the intensity of the lipids (i.e. lipid profile) changes as a function of both sex (red = female, blue = male) and age (young = yellow, old = blue). Furthermore, within each class of lipids, subcategories of lipids are identified that demonstrate increased intensity and decreased intensity as function of age in both sexes. For example, in each row a change of colour from blue in females to red in males along the age axis indicates a faster rate of change with age in males. Similar trends are observed in sex only and age only analysis of the lipids data (see Supplementary Figures S5 and S6).

## Discussion

Female and male *Daphnia magna* are genetically identical and yet show considerable differences in ageing rate and their overall lifespans^37^. Earlier studies using a variety of model organisms have revealed lipids as important contributors to both ageing and lifespan. In this study, we provide the first evidence of the contribution of lipids to ageing in both male and female *Daphnia magna*, finding that more than half of all lipids measured are statistically different between female and male *D. magna* with age.

### Genetically identical female and male *D. magna* show distinct lipid profiles based on sex

The present study clearly demonstrates that sex is the main factor that influences lipid composition in *D. magna* (Fig. 2). Previous studies across a wide range of species have identified similar trends whereby the composition and level of lipids differ between sexes^27,52–54^, with the primary cause linked to differences in reproductive needs and hormone levels between the sexes^53^. Currently, our knowledge regarding molecular mechanisms of hormone regulation in *Daphnia* is limited. However, it has been shown that male *Daphnia* are induced by the juvenile hormone (JH) or a JH pathway-related molecules emitted by the mother in response to environmental conditions, known as environmental sex determination^55–59^. Therefore, the reproductive demands placed on female and male daphnids are extremely different. *Daphnia* reproduce by cyclical parthenogenesis. Under normal conditions with low levels of stress factors, *Daphnia* reproduces asexually and the mother clonally produces daughters. Under stressed conditions, male *Daphnia* are produced for sexual reproduction, leading to production of diapause eggs that remain in arrested development until more favourable reproductive conditions return^60^. As the purpose of male production is for sexual reproduction and sexual reproduction in *Daphnia* is limited to a narrow window of time, males characteristically have much shorter lifespans and smaller body size than females. Therefore, males usually invest less energy into growth and instead the majority of their energy is directed towards maximising egg fertilisation^37^. Therefore, it is possible that the lipidome of *D. magna* separate strongly by sex due in part to the energy demands related to the different reproductive roles.

It is important to highlight that lipids are diverse and play a multitude of roles within biological systems. Therefore, by investigating changes in different classes of lipids between sexes it may provide clues with regards to differences in regulation of biological pathways and needs of both sexes. For example, triglycerides (TG) and diglycerides (DG) are more frequently, but not exclusively, lower in male compared to female *D. magna* (Fig. 4). TGs are the main form of fat recorded in humans and are present across many species. They are found in the blood and act as an energy supply for the body in addition to being stored as body fat when excess is present. The role of TG as the most abundant and accessible energy supply supports the idea that female *D. magna* would require higher concentrations due to the energy demand placed on cyclical parthenogenetic reproduction, growth rate, body size and swimming speed.

Glycerophospholipids appear to show the opposite trend to neutral lipids with phosphatidylethanolamine (PE) and phosphatidylcholine (PC) both observed to be predominantly, but not exclusively, higher in male compared to female *D. magna* (Fig. 4); however overall across both sexes PE and PC largely decline with age (Fig. 6a,b). PEs are an essential component of biological membranes and play a key role in cell and organelle membrane fusion, trafficking and curvature, oxidative phosphorylation, mitochondrial biogenesis and autophagy^61^. In total, PEs account for approximately 25% of all mammalian glycerophospholipids and are particularly enriched in the brain where they account for 45% of total glycerophospholipids and reportedly act as a precursor to the ligand for cannabinoid receptors^62^. In recent years the role of PE in mammalian health has begun to be established, following the discovery of a relationship with Alzheimer’s disease, Parkinson’s disease, non-alcoholic liver disease and virulence of some pathogenic organisms^61^. PE and PC are directly linked as in mammalian liver the enzyme phosphatidylethanolamine *N*-methyltransferase (PEMT) catalyses the conversion of PE to PC by the transfer of three methyl groups from *S*-adenosylmethionine to PE. PC is also a major component of biological membranes and is a pulmonary surfactant whilst also acting as an important membrane cell-signalling molecule^63^. Previous research has reported the decline of PE and PC with age in mouse liver and brain. The impact of this is altered membrane fluidity as membranes become more fluid when containing a low PC: PE ratio, or low cholesterol content. Membrane fluidity has shown the decline with age in rat brain, liver and heart, whilst in the long-lived naked mole rat maintains membrane fluidity through a specific glycerophospholipid/fatty acid saturation profile^64^. The loss of membrane fluidity with age may be in-part due to dyslipidemia observed with age. Investigation in a Japanese population highlighted a link between increased total ester-linked PE and PC species with age, but a decline in specific ether-linked PE and PC species with age^65^. The different behaviour of specific lipid species with age may be linked to the role of each lipid species. In hEP-2 cells, ether-linked PE has an alkenyl group and represents 70–80% of the inner leaflet of the plasma membrane, where-as ether-linked PC species tend to have an alkyl group and represent 70–80% of the outer leaflet of the plasma membrane^66^. The abundance of ether-linked glycerophospholipids indicates their biological importance; however the specific role remains unclear. Possible suggestions include that alkenyl-PE species are the main source of polyunsaturated fatty acids for prostanoid production and cell signalling. Alkenyl-linked glycerophospholipids (plasmalogens) are reportedly active in protection from oxidative damage and alkenyl-PE is essential for cholesterol transport from the cell surface and endocytic compartments to the endoplasmic reticulum^66^. The overall higher relative lipid intensity of PE and PC in males to females (Fig. 6) could possibly be linked to the anti-oxidant role as male *D. magna* show a faster accumulation of DNA damage in real-time^37^ and thus when not considering age may show a higher demand for oxidative protection measures.

In *D. magna*, we observed that sphingomyelin (SM) levels are higher in males. The same trend has been observed with humans whereby females have lower recorded SM^67^. This has been linked to the different distribution of each lipid species and the metabolic consequences associated with that^67^. SM is a major component of the lipid bilayer of cell membranes and so mirrors the observations seen for PE and PC which are also both major lipids present in the membrane lipid bilayer. Interestingly, the more predominant sphingolipid, ceraminde (Cer), is largely lower in male *D. magna*. Cer is considered a centre for sphingolipid metabolism which has been linked to regulation of antiproliferative responses such as growth inhibition, apoptosis, differentiation and senescence^68,69^. Often changes with Cer and SM are linked with ageing, discussed later in this article. Thus it is possible that when investigating sex differences without considering the age of the females and males, we are measuring the overall difference in the level of Cer and SM with age encapsulated by each sex^69^.

### Genetically identical female and male *D. magna* show divergence of lipidome as a function of age

To elucidate age related changes within each sex, changes in the lipid composition of male and female *Daphnia* as a function of age was investigated (Supplementary Tables S5, S6, Fig. 6a,b). Although female *Daphnia magna* reproduce via cyclical parthenogenesis throughout the duration of their lifespan, their reproductive capacity and clutch size declines as they age when they reach 20% of their lifespan (Constantinou *et al*., 2019). Furthermore, the swimming speed also declines continuously with age in female *Daphnia*^37^. The reduced energy demand for reproduction and movement is reflected in the changes observed in the lipid composition of females as a function of age (Supplementary Table S5). A similar trend was also observed among male *D. magna*, whereby the lipid composition and concentrations changed linearly with age (Supplementary Table S5). This could also be linked to decline in swimming speed as a function of age in males. Furthermore, fertilisation success is reduced in both females and males as they age and thus males may demand less energy for reproduction^37^.

As demonstrated in Supplementary Table S5, the lipid composition of 40 days old females (40% of their lifespan) is equally as diverged and distinct from a young sexually mature *Daphnia* (10% of lifespan) as it is from an old *Daphnia* (80% of lifespan). This indicates that lipid composition is continuously altering and evolving with age, reflective of the different needs of an individual as they age. Figure 6b and Supplementary Table S6 demonstrates that males follow the same trend; however, the changes in lipid composition are much more pronounced in the second half of their life. The more pronounced changes in the lipidome of male *Daphnia* at later stages of their life compared to their midlife point could be partly due to the time points that were used for the male samples. In this analysis, we used samples that represent 16%, 20%, 40% and 80% of the lifespan of the male *Daphnia*. Males are not sexually mature until 8 days therefore it was not possible to use a representative sample of 10% of lifespan (5 days) to match the youngest age group for females where aged 10 days is representative of 10% of lifespan.

When considering age for both sexes separately in the analysis (Figs. 6a,b), we identified a higher proportion of TG and DG lipid classes changing with age of which the majority are increasing. Notably, DG shows the bigger gap between those decreasing and increasing in relative intensity in addition to male DG relative intensity increasing with age 1.59-fold more than those seen increasing in females. DG can be metabolised by bacteria, yeast, plants and animals. It is crucial for cell growth and development as it acts as a basis to which components can be added to synthesise complex lipids whilst also acting as a source of free fatty acids^70^. DG acts as a direct precursor for PC, PE, phosphatidic acid (PA) and TG and in doing so has a downstream effect on the production of phosphatidylinositol (PI) and cardiolipins^70^. Given the crucial role of DG, regulation is essential to maintain a permanent reservoir. During the ageing process, lipid dysregulation can occur, often exemplified by humans with the age-associated link to type 2 diabetes, dyslipidemia and altered lipid profiles seen in many cancers. It is possible that age-related dysregulation is occurring in *D. magna* resulting in the reservoir of DG not being utilised efficiently. If true, this may contribute to the majority of PE and PC lipids showing a decline in relative intensity with age across both sexes separately (Fig. 6a,b).

Cholestrol ester (ChE) is a neutral lipid that appears to decline in relative intensity with age in females yet increases in intensity with age in males. ChE is the inactive form of cholesterol and it is primarily used for transport of cholesterol to organs or to act as a biologically inert storage of excess cholesterol by the addition of fatty acids to the hydroxyl group results in less polarity making the lipid ‘inert’^71^. In humans, free cholesterol is esterified by lecithin:cholesterol acyltransferase (LCAT) to form high-density lipoprotein (HDL) particles. In turn, HDL particles can bind to hepatocytes to uptake ChE from HDL in to the liver where it can be hydrolysed by cholesterol ester hydrolase to either be converted back to free cholesterol or turned into bile acids. Alternatively, ChE can be exchanged from HDL for TG which can influence composition, size and ultimately function of lipoproteins^72^. Currently there is limited information available about how cholesterol esters levels change during ageing or between sexes. However, the altered activity of LCAT and HDL levels have been widely investigated in association with age-related diseases^72–75^. Furthermore, it has been demonstrated that high levels of SM inhibit the activity of LCAT. In humans, SM level is reportedly lower in males compared to females, exposing females to greater risk of LCAT suppression which could explain differences in the rate of age-associated diseases between sexes^67^. HDL particles have many positive effects such as anti-inflammatory, anti-oxidant, anti-apoptotic, anti-thrombotic and vasodilation^72^. If conversion of cholesterol to ChE is limited with ageing in female *D. magna* by altered HDL efficacy, it is possible that reduced HDL efficacy contributes to the observed increase in oxidative damage observed in female *D. magna* with age^37^. Given that some lipids are more prone to oxidative damage it is likely that the difference in lipid composition between male and female *D. magna* contributes to observed differences in age-related DNA damage. Lower HDL-Cholesterol in males has been reported to associate with lower SM concentrations compared to females, resulting in males having an increased cardiovascular risk^67^.

### Genetically identical female and male *D. magna* show different rates of change in relative lipid intensities with age between sexes

Changes in lipid composition as a function of sex and age interaction was also investigated (Fig. 7). The neutral lipids TG and DG both show a stronger relationship with age in males compared to females. Given that the lifespan of a male *Daphnia* is half the lifespan of a female *Daphnia*^37^, it is plausible that the faster rate of change in certain lipids is to account for/contributes to the observed shorter lifespan in males. However, this idea does not hold true for glycerophospholipids PE and PC as they demonstrate a more balanced relationship between faster and slower rates of change in males compared to females, moderately biased towards slower rate. A slower rate of change in males based on age in days indicates a dramatically slower rate as a factor of proportional longevity. As previously discussed, the relative intensity of PE and PC largely decline with age. A slower rate of change in males indicates that this loss is occurring at a slower pace compared to females. Given that DG, a precursor to PE and PC, shows a much higher increase in relative intensity with age in males (1.59-fold more than females), it bodes well for the continued production of PE and PC from the DG reserve through-out the lifespan of a male *Daphnia*.

Sphingolipids SM and Cer show the opposite trend to neutral lipids, displaying a greater bias towards changing lipid intensity at a much slower rate in males compared to females. Sphingolipids have been linked to many functions and diseases influenced by the ageing process, including immune response, inflammation, cancer, metabolic and cardiovascular diseases and neurodegeneration. Ceramide induces cellular senescence, a mode of suspended cell division which has positive tumour suppressor effects as well as negative impacts on cardiovascular system, metabolic and immune systems^69^. Senescence has been shown to contribute to ageing and may partially explain the association of sphingolipids with age-associated conditions. In humans, SM can be degraded to produce Cer and PC and reversely, PC and Cer can be utilised to produce SM and DG^69^. These interactions between various lipid groups create a complex and sensitive feedback loop constantly shifting and moderating in response to lipid alterations. The interaction effect in male *D. magna* of SM and Cer is likely linked to alterations observed in DG and PC.

Finally, Fig. 8 highlights clustering of lipids altered in sex:age interaction is based largely on sex followed closely by age. The interaction of sex and age encapsulates approximately half of changes to lipid intensity observed in sex and age (Fig. 3a). To conclude, in this study we have demonstrated, for the first time, profound differences in the lipid composition of male and female *Daphnia magna*. We have also demonstrated marked shifts in lipid composition with age, indicating a possible relationship between *Daphnia* lipid composition and age-related processes.

## Supplementary information

Supplementary information

Supplementary information 2

Supplementary information 3

## Data Availability

The datasets generated and analysed during the current study are available from the corresponding authors on reasonable request.
