# Supplementary material for: Characterisation of the dynamic nature of lipids throughout the lifespan of genetically identical female and male Daphnia magna
Source: Sci Rep. 2020 Mar 27;10:5576. doi: 10.1038/s41598-020-62476-z (PMC7101400; doi:10.1038/s41598-020-62476-z)
Supplement: Supplementary file 1 — Supplementary information [file 41598_2020_62476_MOESM1_ESM.docx]

**Title: Characterisation of the dynamic nature of lipids throughout the lifespan of genetically identical female and male *Daphnia magna*: Supplementary Data S2 – MS/MS Fragmentation Spectra**

Authors: Julia K. Constantinou*^1^, Andrew D. Southam ^1,2^, Jouni Kvist ^3^, Martin R. Jones ^1,4^, Mark R. Viant ^1,2^, Leda Mirbahai*^5^

^1^ School of Biosciences, University of Birmingham, Birmingham, B15 2TT, UK

^2^ Phenome Centre Birmingham, University of Birmingham, Birmingham, B15 2TT, UK

^3^ Research Program for Molecular Neurology, University of Helsinki, Haartmaninkatu 8, 00014, Finland

^4^ Eawag, Swiss Federal Institute of Aquatic Science and Technology, Dübendorf, Switzerland

^5^ Warwick Medical School, University of Warwick, Coventry, CV4 7AL, UK

* Corresponding authors:

Julia Constantinou: JKC335@student.bham.ac.uk

Leda Mirbahai: [Leda.Mirbahai@warwick.ac.uk](mailto:Leda.Mirbahai@warwick.ac.uk)

**Section A: Figures and Tables**

**Supplementary Figure S1a:** Female dry weight per individual *Daphnia* aged 10 to 40 days with linear line of fit.

**Supplementary Figure S1b:** Female dry weight per individual *Daphnia* aged 40 to 80 days with linear line of fit.

| **Predicted weights at intermediate ages** | | | | | |  |
| --- | --- | --- | --- | --- | --- | --- |
| **Age** | **Predicted Weight (g)** | **No. daph required to approx. 5xFD80 sample** | | | **Animals required per rep** | **Total weight of replicate (g)** |
| **Using Supplementary Figure S1a** | | | | | | |
| 15 | 0.00036 | 0.004435 / 0.00036 | | 12.31944 | 12 | 0.00432 |
| 25 | 0.00056 | 0.004435 / 0.00056 | | 7.919643 | 8 | 0.00448 |
| 30 | 0.00066 | 0.004435 / 0.00066 | | 6.719697 | 7 | 0.00462 |
| 35 | 0.00076 | 0.004435 / 0.00076 | | 5.835526 | 6 | 0.00456 |
| **Using Supplementary Figure S1b** | | | | | | |
| 45 | 0.000845 | 0.004435 / 0.000845 | | 5.248521 | 5 | 0.004225 |
| 50 | 0.00085 | 0.004435 / 0.00085 | | 5.217647 | 5 | 0.00425 |
| 55 | 0.000855 | 0.004435 / 0.000855 | | 5.187135 | 5 | 0.004275 |
| 60 | 0.00086 | 0.004435 / 0.00086 | | 5.156977 | 5 | 0.0043 |
| 65 | 0.000865 | 0.004435 / 0.000865 | | 5.127168 | 5 | 0.004325 |
| 70 | 0.00087 | 0.004435 / 0.00087 | | 5.097701 | 5 | 0.00435 |
| 75 | 0.000875 | 0.004435 / 0.000875 | | 5.068571 | 5 | 0.004375 |
| **Age in days** | | | **Dry Weight per single *Daphnia* (actual)** | |  |  |
| 10 | | | 0.000233 | |  |  |
| 20 | | | 0.000479 | |  |  |
| 40 | | | 0.000827 | |  |  |
| 80 | | | 0.000887 | |  |  |

**Supplementary Table S1:** Actual and predicted dry mass of female *Daphnia magna* age groups. Female *D.magna* aged 80 days have an average weight of 0.000887g but each biological replicate contains 5 daphnia therefore giving an average total replicate weight of 0.004435g.

**Supplementary Figure S2a:** Male dry weight per individual *Daphnia* aged 10 to 20 days with linear line of fit.

**Supplementary Figure S2b:** Male dry weight per individual *Daphnia* aged 20 to 40 days with linear line of fit.

| **Predicted weights at intermediate ages** | | | | |  |
| --- | --- | --- | --- | --- | --- |
| **Age** | **Predicted Weight (g)** | **No. daph required to approx. 5xFD80 sample** | | **Animals required per rep** | **Total weight of replicate (g)** |
| **Using Supplementary Figure S2a** | | | | | |
| 8 | 0.000087 | 0.004435 / 0.000087 | 50.97701 | 51 | 0.004437 |
| 13 | 0.000137 | 0.004435 / 0.000137 | 32.37226 | 32 | 0.004384 |
| 15 | 0.000157 | 0.004435 / 0.000157 | 28.24841 | 28 | 0.004396 |
| 18 | 0.000187 | 0.004435 / 0.000187 | 23.71658 | 24 | 0.004488 |
| **Using Supplementary Figure S2b** | | | | | |
| 23 | 0.000223 | 0.004435 / 0.000223 | 19.88789 | 20 | 0.00446 |
| 25 | 0.000225 | 0.004435 / 0.000225 | 19.71111 | 20 | 0.0045 |
| 28 | 0.000228 | 0.004435 / 0.000228 | 19.45175 | 19 | 0.004332 |
| 30 | 0.00023 | 0.004435 / 0.00023 | 19.28261 | 19 | 0.00437 |
| 33 | 0.000233 | 0.004435 / 0.000233 | 19.03433 | 19 | 0.004427 |
| 35 | 0.000235 | 0.004435 / 0.000235 | 18.87234 | 19 | 0.004465 |
| 38 | 0.000238 | 0.004435 / 0.000238 | 18.63445 | 19 | 0.004522 |

| **Age in days** | **Dry Weight per single *Daphnia* (actual)** |
| --- | --- |
| 10 | 0.000107 |
| 20 | 0.000207 |
| 40 | 0.000233 |

**Supplementary Table S2:** Actual and predicted dry mass of male *Daphnia magna* age groups. Female *D.magna* aged 80 days have an average weight of 0.000887g but each biological replicate contains 5 daphnia therefore giving an average total replicate weight of 0.004435g.


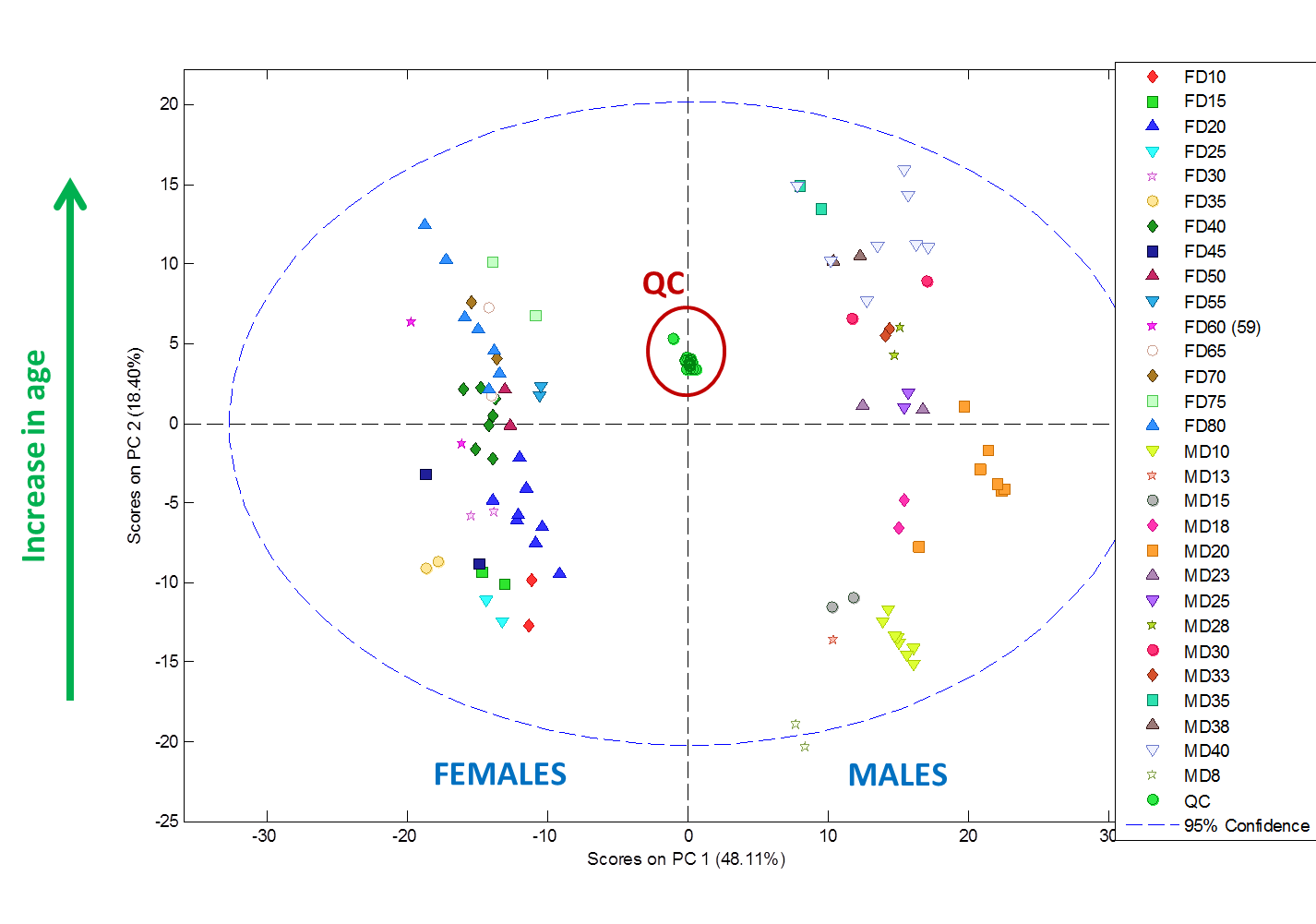


**Supplementary Figure S3: Principal component analysis of normalised positive ion mode lipidomics data for male and female *Daphnia magna* with QC samples shown.** Principal component 1 shows clear separation based on sex with females grouped to the left and males to the right. Principal component 2 shows separation by age depicted by colour and symbol. Female *D. magna* age range is 10 days to 80 days (maximum sampled age = 80 days). For female age groups 20, 40 and 80 days, eight technical replicates were used and for intermediate ages two technical replicates were used. Male *D. magna* age groups ranging from 8 days to 40 days were used (maximum sampled age = 40 days). For male *D. magna* age groups 10, 20 and 40 days, eight technical replicates were used and for intermediate age groups two technical replicates were used. For detailed information regarding biological replicate numbers see Supplementary Table S1 and Supplementary Table S2. QC samples are green circles highlighted by the red circle and QC label.


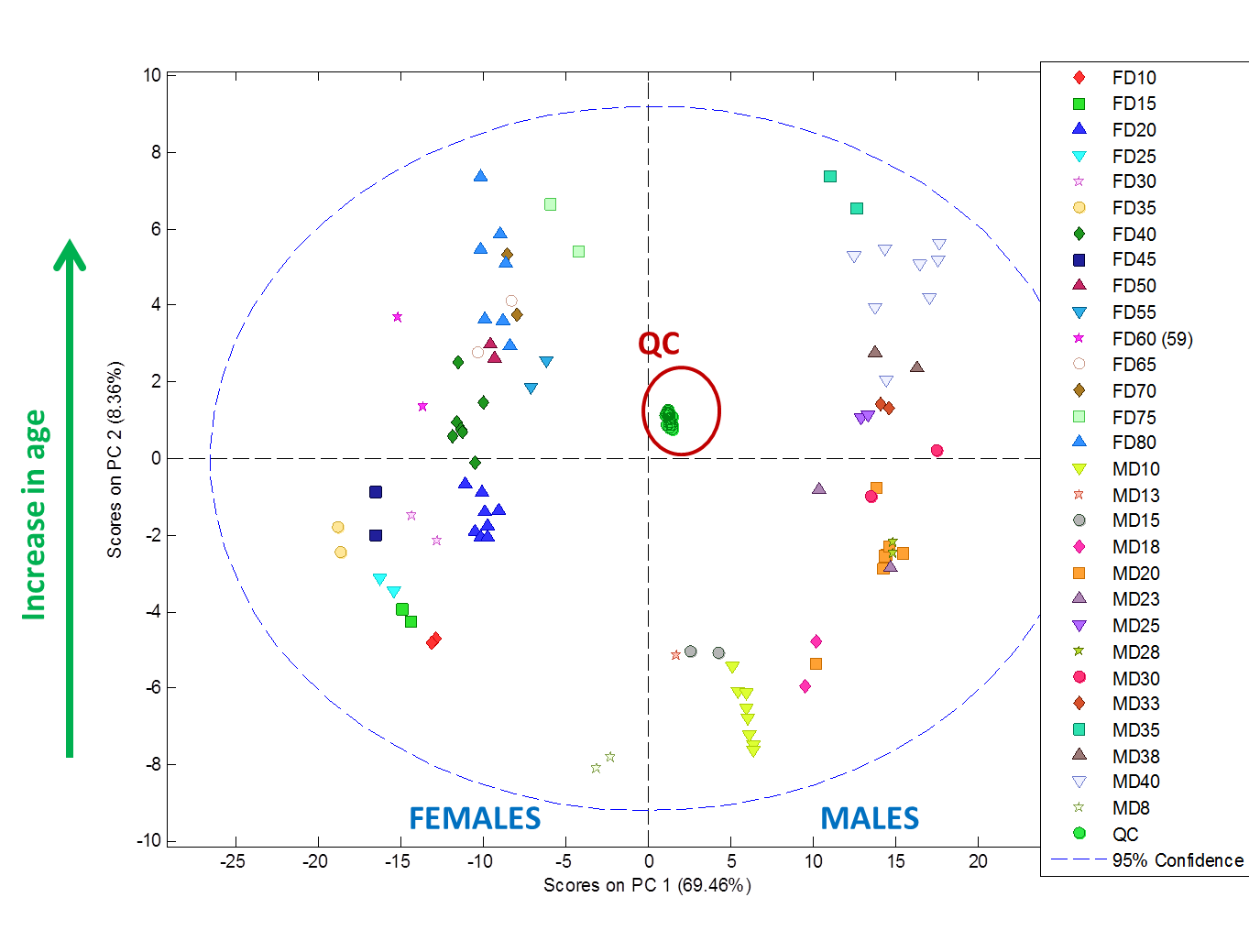


**Supplementary Figure S4: Principal component analysis of normalised negative ion mode lipidomics data for male and female *Daphnia magna* with QC samples shown.** Principal component 1 shows clear separation based on sex with females grouped to the left and males to the right. Principal component 2 shows separation by age depicted by colour and symbol. Female *D. magna* age range is 10 days to 80 days (maximum sampled age = 80 days). For female age groups 20, 40 and 80 days, eight technical replicates were used and for intermediate ages two technical replicates were used. Male *D. magna* age groups ranging from 8 days to 40 days were used (maximum sampled age = 40 days). For male *D. magna* age groups 10, 20 and 40 days, eight technical replicates were used and for intermediate age groups two technical replicates were used. For detailed information regarding biological replicate numbers see Supplementary Table S1 and Supplementary Table S2. QC samples are green circles highlighted by the red circle and QC label.

**Supplementary Table S5: Comparison of lipids showing significant change in levels between female D. magna age groups.** Significance determined by adjusted *p*-value <0.001. Reference age group set at 10 20, 40 and 80 days respectively. Lipids highlighted within the table represent those present in the reference group but not in the comparison group (e.g. 10:20 shows 87 significantly changing lipids present in aged 10 days but not aged 20 days, 10:20 verses 10:40 shows 49 lipids unique to aged 10 days not shared with aged 20 or 40 days). ‘Significant across all group comparisons’ represents lipids significantly changing levels in the reference group only, not shared with any other age. ‘Non-significant’ represent lipids identified in lipidomics analysis not shown to change in level >0.001 adjusted *p-*value. ‘Total significant lipids’ shown in table represent the total number of lipids significantly changing level highlighted in the cross comparison table. ‘Total lipids’ represent the total number of lipids identified during analysis and is always equal to 2556.

**Supplementary Table S6: Comparison of lipids showing significant change in levels between male D. magna age groups.** A Significance determined by adjusted *p*-value <0.001. Reference age group set at 8, 10 20 and 40 days respectively. Lipids highlighted within the table represent those present in the reference group but not in the comparison group (e.g. 8:10 shows 34 significantly changing lipids present in aged 8 days but not aged 10 days, 8:10 verses 8:20 shows 24 lipids unique to aged 8 days not shared with aged 10 or 20 days). ‘Significant across all group comparisons’ represents lipids significantly changing levels in the reference group only, not shared with any other age. ‘Non-significant’ represent lipids identified in lipidomics analysis not shown to change in level >0.001 adjusted *p-*value. ‘Total significant lipids shown in table’ represent the total number of lipids significantly changing level highlighted in the cross comparison table. ‘Total lipids’ represent the total number of lipids identified during analysis and is always equal to 2556.

| Abbreviation | Lipid Class Description | Lipid Class Classification |
| --- | --- | --- |
| AcCa | Acyl Carnitine | Fatty acyl and other lipids |
| Cer | Ceramide | Sphingolipids |
| ChE | Cholesterol ester | Neutral lipids, sterols |
| CL | Cardiolipin | Phospholipids |
| DG | Diglyceride | Neutral lipids, sterols |
| FA | Fatty acid | Fatty acyl and other lipids |
| GD1a | Ganglioside, disialo tetrahexosyl ceramide | Sphingolipids |
| GT3 | Ganglioside | Sphingolipids |
| Hex1Cer | Hexosyl ceramide | Sphingolipids |
| Hex2Cer | Dihexosyl ceramide | Sphingolipids |
| LPC | Lyso phosphatidylcholine | Phospholipids |
| LPE | Lyso phosphatidylethanolamine | Phospholipids |
| MG | Monoglyceride | Neutral lipids, sterols |
| MGDG | Monogalactosyl diacylglycerol | Glycoglycerolipids (plants) |
| MLCL | Monolyso cardiolipin | Phospholipids |
| OAHFA | OAcyl(gammahydroxy) FA | Fatty acyl and other lipids |
| PC | Phosphatidylcholine | Phospholipids |
| PE | Phosphatidylethanolamine | Phospholipids |
| PG | Phosphatidylglycerol | Phospholipids |
| phSM | Sphingomyelin, phytosphingosine | Sphingolipids |
| PI | Phosphatidylinositol | Phospholipids |
| PIP | Phosphatidylinositol phosphate | Phospholipids |
| Pme | Phosphatidylmethanol | Fatty acyl and other lipids |
| PS | Phosphatidylserine | Phospholipids |
| SM | Sphingomyelin | Sphingolipids |
| SPH | Sphingosine bases | Sphingolipids |
| TG | Triglyceride | Neutral lipids, sterols |
| WE | Wax esters (fatty acid esters) | Fatty acyl and other lipids |
| ZyE | Zymosterol ester | Neutral lipids, sterols |

**Supplementary Table S7: Lipid class abbreviations, descriptions and classifications.**


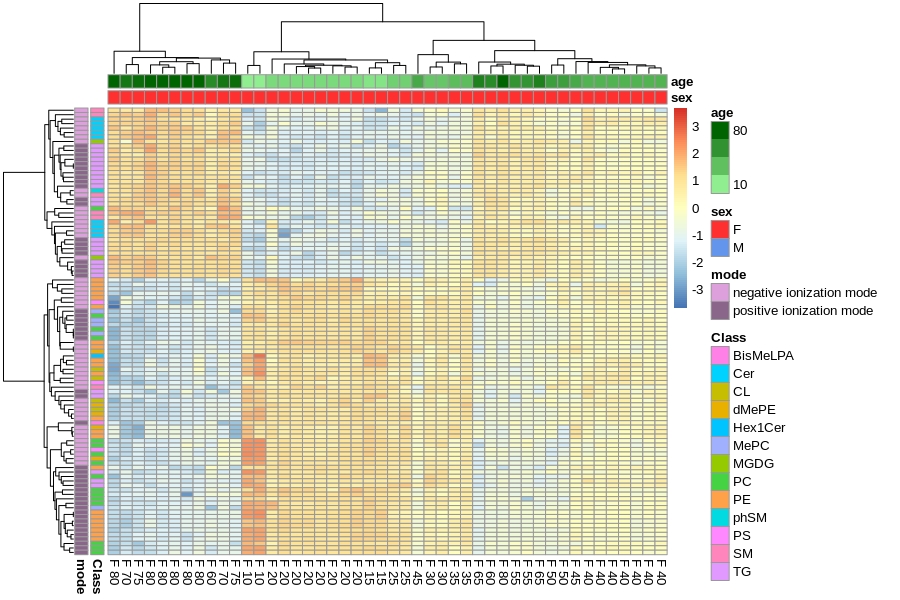


**Supplementary Figure S5:** **Heatmap of lipid changes with age in female *D. magna* only.** Heatmap of lipidomics analysis for top 50 lipids significantly-changing lipid intensities in positive ion mode (dark purple) and top 50 lipids significantly changing levels in negative ion mode (light purple, adjusted *p­*-value <0.001) in female *D. magna* with age. The heat map shows 100 selected lipids indicated by class colours (rows) and 29 samples (columns). Colours correspond to per-lipid z-score that is computed from area under the curve. Lipids and samples were hierarchically clustered based on Euclidean cluster extraction and average linkage.


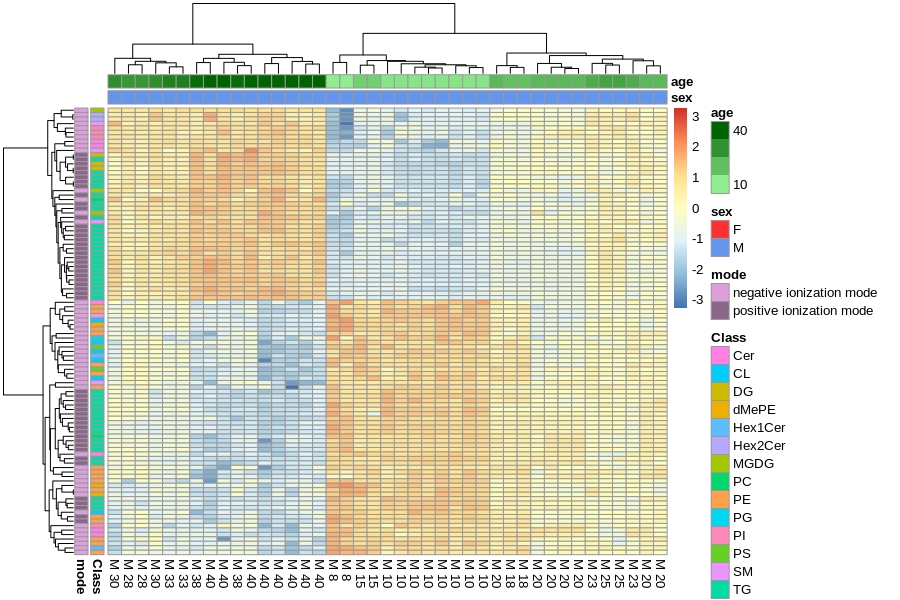


**Supplementary Figure S6:** **Heatmap of lipid changes with age in male *D. magna* only.** Heatmap of lipidomics analysis for top 50 lipids significantly-changing lipid intensities in positive ion mode (dark purple) and top 50 lipids significantly changing levels in negative ion mode (light purple, adjusted *p­*-value <0.001) in male *D. magna* with age. The heat map shows 100 selected lipids indicated by class colours (rows) and 29 samples (columns). Colours correspond to per-lipid z-score that is computed from area under the curve. Lipids and samples were hierarchically clustered based on Euclidean cluster extraction and average linkage.

**Section B: MS/MS Fragmentation Spectra**

Contents

[MS/MS identities of Table 1 in main paper 14](#_Toc25844711)

[Example peaks from each class 24](#_Toc25844712)

[Acylcarnitines 24](#_Toc25844713)

[Ceramides 25](#_Toc25844714)

[Cholesterol Esters 26](#_Toc25844715)

[Diglycerides 27](#_Toc25844716)

[Lysophospholipids 28](#_Toc25844717)

[Phosphatidylcholines 29](#_Toc25844718)

[Phosphatidylethanolamines 30](#_Toc25844719)

[Phosphatidylglycerols 31](#_Toc25844720)

[Phosphatidylinositol 32](#_Toc25844721)

[Sphingomyelines 34](#_Toc25844722)

[Triglycerides 35](#_Toc25844723)

# MS/MS identities of Table 1 in main paper

P2118; positive ion; TG(32:3)+NH4; TG(2:0_14:0_16:3)+NH4; 594.47282m/z; RT10.43 min

Loss of 2:0+NH_3_

Loss of 14:0+NH_3_

Loss of 16:3+NH_3_

P2141; positive ion; TG(34:3)+NH4; TG(2:0_16:0_16:3)+NH4; 622.50412 m/z; RT11.61 min

Loss of 16:0+NH_3_

Loss of 2:0+NH_3_

Loss of 16:3+NH_3_

P2875; positive ion; TG(50:3)+NH4; TG(16:0_16:0_18:3)+NH4; 846.7545215m/z; RT18.13 min

Loss of 18:3+NH_3_

Loss of 16:0+NH_3_

P645; positive ion; MePC(30:4p)+NH4; 713.52282m/z; RT7.95 min

Fragment peak indicative of phosphatidylcholine

P130; positive ion; ChE(16:1)+NH4; 640.60271m/z; RT20.55 min

Fragment peak indicative of cholesterol ester.

P136; positive ion; ChE(18:1)+NH4; 668.63401m/z; RT21.56 min

Fragment peak indicative of cholesterol ester.

P133; positive ion; ChE(17:1)+NH4; 654.61836m/z; RT21.07 min

Fragment peak indicative of cholesterol ester.

P2029; positive ion; SM(t40:2)+H; 801.64802m/z; RT12.20 min

Fragment peak indicative of sphingomyelin

P1947; positive ion; SM(d34:3)+H; 699.543537m/z; RT8.92 min

Fragment peak indicative of sphingomyelin

AN1167; negative ion; SM(t40:2)+HCOO; 845.63894m/z; RT12.19 min

Loss of HCOO adduct plus methyl group. Indicative of sphingomyelin.

# Example peaks from each class

## Acylcarnitines

P43; 422.32649m/z; RT2.46 min; AcCa(18:3)+H

Characteristic acylcarnitine fragment

P25; 400.34214m/z; RT3.95 min; AcCa(16:0)+H

Characteristic acylcarnitine fragment

## Ceramides

P113; positive ion; Cer(d18:1_16:0)+H; 538.51937m/z; RT11.48 min

Sphingoid d18:1 base

P108; positive ion; Cer(d16:1_16:0)+H; 510.48807m/z; RT10.34 min

Sphingoid d16:1 base

## Cholesterol Esters

P130; positive ion; ChE(16:1)+NH4; 640.60271m/z; RT20.55 min

Fragment peak indicative of cholesterol ester.

P136; positive ion; ChE(18:1)+NH4; 668.63401m/z; RT21.56 min

Fragment peak indicative of cholesterol ester.

## Diglycerides

P372; 630.5092m/z; RT10.33 min; DG(18:3_18:3)+NH4

Monoglyceride 18:3

P294; 608.52485m/z; RT11.89 min; DG(16:0_18:3)+NH4

Monoglyceride 16:0

Monoglyceride 18:3

## Lysophospholipids

AN339; 540.3307m/z; RT3.80 min; LPC(16:0)+HCOO

16:0 fatty acid

AN376; 474.26262m/z; RT2.37; LPE(18:3)-H

18:3 fatty acid

## Phosphatidylcholines

AN512; 804.57601m/z; RT11.60 min; PC(16:0_18:1)+HCOO

18:1 fatty acid

16:0 fatty acid

AN579; 824.54471m/z; RT9.36 min; PC(18:3_18:2)+HCOO

18:2 fatty acid

18:3 fatty acid

## Phosphatidylethanolamines

AN749; 740.52358m/z; RT11.08 min; PE(18:1_18:2)-H

18:1 fatty acid

18:2 fatty acid

AN741; 742.53923m/z; RT11.88 min; PE(18:1_18:1)-H

18:1 fatty acid

## Phosphatidylglycerols

AN890; 743.48686m/z; RT9.48 min; PG(16:0_18:3)-H

16:0 fatty acid

18:3 fatty acid

AN900; 765.47121m/z; RT8.22 min; PG(18:3_18:3)-H

18:3 fatty acid

## Phosphatidylinositol

AN927; 857.51856m/z; RT9.30 min; PI(18:1_18:3)-H

241 peak indicative of phosphatidylinositol

18:1 fatty acid

18:3 fatty acid

AN928; 857.51856m/z; RT9.82 min; PI(16:0_20:4)-H

20:4 fatty acid

16:0 fatty acid

241 peak indicative of phosphatidylinositol

## Sphingomyelines

P2029; positive ion; SM(t40:2)+H; 801.64802m/z; RT12.20 min

Fragment peak indicative of sphingomyelin

P1947; positive ion; SM(d34:3)+H; 699.543537m/z; RT8.92 min

Fragment peak indicative of sphingomyelin

AN1167; negative ion; SM(t40:2)+HCOO; 845.63894m/z; RT12.19 min

Loss of HCOO adduct plus methyl group. Indicative of sphingomyelin.

## Triglycerides

P2118; positive ion; TG(32:3)+NH4; TG(2:0_14:0_16:3)+NH4; 594.47282m/z; RT10.43 min

Loss of 2:0+NH_3_

Loss of 14:0+NH_3_

Loss of 16:3+NH_3_

P2141; positive ion; TG(34:3)+NH4; TG(2:0_16:0_16:3)+NH4; 622.50412 m/z; RT11.61 min

Loss of 16:0+NH_3_

Loss of 2:0+NH_3_

Loss of 16:3+NH_3_

P2875; positive ion; TG(50:3)+NH4; TG(16:0_16:0_18:3)+NH4; 846.7545215m/z; RT18.13 min

Loss of 18:3+NH_3_

Loss of 16:0+NH_3_
